# Children's and adolescents' expectations, evaluations and reasoning about a bystander who challenges social exclusion within intragroup and intergroup peer contexts

**DOI:** 10.1111/bjdp.12522

**Published:** 2024-09-21

**Authors:** Ayşe Şule Yüksel, Sally B. Palmer, Eirini K. Argyri, Adam Rutland

**Affiliations:** ^1^ Department of Psychology University of Exeter Exeter UK; ^2^ Graduate School of Education University of Exeter Exeter UK

**Keywords:** adolescents, bystander challenging, children, intergroup, intragroup

## Abstract

This paper examined British children's (8‐ to 10‐year‐olds) and adolescents' (13‐ to 15‐year‐olds, *N* = 340; Female *N* = 171, 50.3%) expectations, evaluations and reasoning about a bystander peer who challenges the social exclusion of an immigrant or non‐immigrant peer by a peer group of non‐immigrant students. Participants read a hypothetical scenario in which a peer was excluded from an afterschool club by the peer group. The scenarios were either intergroup or intragroup contexts. Participants' expectations of a peer bystander challenging the social exclusion by the peer group, their perception of how the peer group would evaluate the challenger, and their reasoning around their expectations were measured. Adolescents were less likely to expect a peer bystander to challenge exclusion compared to children. Participants' perceptions of how the group would evaluate the challenger were significantly lower in intergroup compared to intragroup contexts. In intergroup contexts, adolescents with low expectations of challenging favoured group dynamics and group repercussions reasoning over moral reasoning, while children did not use group repercussions reasoning.

**Statement of Contribution**

**What is already known on this subject?**

- Youth who challenge bullying as a bystander can help reduce it.
- Yet peers often do not challenge social exclusion as a bystander, especially in intergroup contexts.
- Adolescents often do not think their ingroup peers will challenge intergroup name calling as a bystander.

**What does the present study add?**

- It examined adolescents’ and children’s expectations, evaluations and reasoning about bystander challenging in either intergroup or intragroup social exclusion contexts.
- Showed adolescents were less likely to expect a peer bystander to challenge social exclusion compared to children.
- Found differences in the social and moral reasoning of children and adolescents within intergroup contexts.

## BACKGROUND

Intergroup exclusion is when someone from one group is excluded by someone from another group (e.g., a non‐immigrant child excluding an immigrant child) because of their social group membership, and it typically derives from prejudicial attitudes and can have harmful psychological and social consequences (Killen, Mulvey, & Hitti, 2013; Killen & Rutland, 2011; Russell et al., 2012). Intragroup exclusion, however, is when an individual is excluded by someone from their own group (e.g., a non‐immigrant child excluding another non‐immigrant child). When youth challenge exclusion as a bystander, they can help minimize it (Evans et al., 2014; Palmer & Abbott, 2018; Polanin et al., 2012), but often peers do not challenge as a bystander (Hawkins et al., 2001). Indeed, research suggests bystander challenging is uncommon within intergroup peer contexts involving bias‐based bullying (e.g., social aggression: Gönültaş & Mulvey, 2020; Palmer et al., 2015).

This study will for the first time directly compare children's and adolescents' expectations, evaluations and reasoning about bystander challenging within both intergroup and intergroup peer contexts. In intragroup peer contexts, one needs to consider group dynamics (i.e., relations between peers within groups), and how bystanders might be evaluated within the group. Intergroup peer contexts require one to both take into account group dynamics and intergroup relations between social identity groups, namely whether these groups vary in terms of social status and are distinctive from each other (Palmer et al., 2021; Rutland & Killen, 2015). Youth expectations, evaluations and reasoning about bystander challenging in peer group contexts are important since they often relate to whether they actually challenge as a bystander when they witness social exclusion (Mulvey et al., 2018).

### Challenging in intergroup and intragroup contexts

The Social Reasoning Developmental approach to social exclusion (SRD; Killen & Rutland, 2011; Rutland et al., 2010) suggests that youth's reluctance to challenge exclusion in intergroup contexts might be related to both intragroup (i.e., fitting in with the peer group) and intergroup concerns (i.e., supporting the ingroup against the outgroup and maintaining positive group distinctiveness). Both of these concerns are salient for intergroup contexts, compared to intragroup contexts, which only involve within‐group considerations. Furthermore, individuals may justify the social exclusion of others and their lack of bystander challenge by focusing on these group‐related rather than moral concerns (e.g., victim welfare or prejudice). Research suggests this focus on group‐related concerns is most likely among adolescents compared to children since understanding of group dynamics and intergroup processes increases with age from childhood into adolescence (Gönültaş et al., 2022; Killen & Rutland, 2011; Mulvey & Killen, 2016). In intergroup contexts, adolescents increasingly consider what the group thinks when making decisions about their own or others' actions within the peer context. Challenging an ingroup norm (i.e., group‐based expectations for behaviour) could impact both how the ingroup is perceived in relation to the outgroup and how the challenger is viewed within the peer ingroup (Gönültaş et al., 2022; Mulvey et al., 2016; Mulvey & Killen, 2016). In intragroup contexts, only the latter would be considered.

To date, only one study has examined adolescents' expectations of peer bystanders challenging in an intergroup context (Mulvey et al., 2016). This study presented a context of ingroup members making race‐based jokes about outgroup members. They found that older adolescents (10th grade) were less likely to expect their ingroup peers to intervene as a bystander than younger adolescents (8th grade). This was due to increased awareness of intergroup processes and group repercussions (i.e., being excluded from the peer group). The present study extends this work by additionally examining; (1) whether children are more likely than adolescents to expect peer bystanders to challenge, given children typically focus less on group‐related concerns; (2) whether expectations of challenging and perceptions of how the group will evaluate challenging differ within intragroup contexts (driven by group dynamics concerns) versus intergroup contexts (driven by both group dynamics and intergroup concerns) and (3) whether developmental and contextual differences are evident when examining peer‐based social exclusion, not relational aggression, since the former is not always seen as a moral transgression (Rutland & Killen, 2015).

### Individual and perceived group evaluations of challenging

SRD research has examined the relations between children's and adolescents' individual and perceived group evaluations in intergroup contexts (Gönültaş et al., 2022; McGuire et al., 2019; Mulvey et al., 2014b; Mulvey & Killen, 2016; Rutland et al., 2015). Findings show that between late childhood and early adolescence, individuals show a stronger differentiation between their own perspective and what they perceive their group's perspective to be. Across this age range, individual evaluations are mostly focused on moral concerns (i.e., “I think we should share fairly or challenge stereotyping”), whereas with age they think their group will be less focused on moral concerns relative to group‐based concerns (i.e., “My group won't like someone who doesn't fit into the group”). What is not known, however, is if this distinction between individual and group evaluations and the relative difference in consideration of moral or group‐based concerns is evident into mid‐adolescence across both intragroup and intergroup contexts of social exclusion. In the current study, we expected that participants would individually evaluate bystanders who challenge exclusion more positively than they think their group would, especially in intergroup contexts.

### Social‐moral reasoning

This study also examined participants' reasoning around their expectations of bystander challenging towards exclusion. Participants' reasoning justifications were coded using categories from Social Domain Theory (Smetana, 2013; Turiel, 2007) and previous SRD research (Killen, Mulvey, & Hitti, 2013; Mulvey et al., 2016; Palmer et al., 2015). The SRD model indicates that youth in peer group contexts weigh up different concerns when they are making social and moral evaluations (Mulvey et al., 2016; Palmer et al., 2015). These concerns are (1) moral (fair and equal treatment of others), (2) group (group identity, group dynamics, intergroup relations) and (3) personal (autonomy and personal preferences).

The SRD model expects that the ability to coordinate and consider multiple concerns across domains increases from childhood into adolescence. For example, children often regard intergroup exclusion as wrong and reject it due to moral concerns (Killen et al., 2001; Rutland & Killen, 2015). Into adolescence, however, an individual's comprehension of group dynamics and intergroup processes increases, which means they can understand that, in some circumstances, intergroup exclusion may be judged as relatively acceptable due to group and personal concerns (Hitti, 2014; Rutland & Killen, 2015). It was expected that participants' reasoning would vary as a function of expectations regarding how likely it would be that a peer bystander who wants to challenge the exclusion will do so. According to SRD, participants who report a high expectation should refer to moral reasons more than group and personal reasons, whereas participants who report low expectation should refer to group and personal reasons more than moral reasons with age. It was an open question as to whether reasoning would differ based on the group context (i.e., intergroup vs. intragroup).

### The present study

With ongoing migration, schools globally are becoming increasingly diverse. In the United Kingdom, approximately 10% of state‐funded school students are born outside of the United Kingdom, and the percentage increases in diverse areas (Briggs, 2019). Research shows that immigrants are likely to encounter bias‐based bullying and exclusion in schools (Xu et al., 2020). In the present study, we examine the timely topic of immigrant social exclusion among school‐age youth. We presented children and adolescents with an imaginary scenario in which a newcomer is excluded by a peer group, but a bystander peer wants to challenge the social exclusion (“challenger”). Participants were asked about their expectations of the challenger peer challenging the social exclusion in either an intergroup or intragroup context. We also asked for evaluations of the bystander once they challenge the exclusion, both from the perspective of the participant (i.e., individual) and the peer group (i.e., perceived group). In the intergroup context, the comparison between the victim's and excluder's group membership was made salient (i.e., British peers excluded an immigrant victim or immigrant peers excluded a British victim). In the intragroup context, the comparison was not made salient (i.e., British peers excluded a British victim or immigrant peers excluded an immigrant victim).

Asking youth about their expectations of a peer bystander who wants to challenge group‐based social exclusion can give an important insight into how participants personally evaluate group norms. This is because it minimizes the possible influence of social desirability that could be more likely when indicating personal decisions about bystander responses. Examining expectations has been considered an authentic reflection of their own bystander intervention (Mulvey et al., 2016; Palmer et al., 2021).

We uniquely examine both children's (aged 8–10) and adolescents' (aged 13–15) expectations of a bystander challenging their peer group, together with participants' individual and perceived peer group evaluations of challenging the exclusion. These age groups were selected as previous research shows a developmental shift from childhood into adolescence whereby adolescents, compared to children, are more likely to consider group‐related concerns when evaluating social exclusion (Killen, Rutland, et al., 2013; Mulvey et al., 2014a) and are less likely to think bystanders will challenge their peer group during intergroup contexts (Mulvey et al., 2016; Palmer et al., 2015). We additionally explore reasoning around these expectations, which can help shed light on the underlying social cognitive processes that inform evaluations and decision‐making (Palmer et al., 2021).

Research has also shown that bystander intentions to challenge the bullying of immigrant outgroup members can increase when they have high levels of intergroup contact (Abbott & Cameron, 2014). Higher levels of intergroup contact can reduce children's prejudice towards outgroups, that is, immigrants (Titzmann et al., 2015), and their evaluations regarding exclusion can become more negative (Crystal et al., 2008; Park et al., 2019). In the current study, therefore, we measured participants' intergroup contact with immigrants to use as a covariate in the analyses.

### Hypotheses

Hypothesis 1 Compared to children, adolescents would be less likely to expect a bystander peer to challenge social exclusion, especially in intergroup contexts compared to intragroup contexts.

Hypothesis 2 Compared to children, adolescents would be more likely to refer to group and personal concerns than moral concerns when reasoning about their expectations of bystander challenging.

Hypothesis 3 Participants who report a higher expectation of the bystander challenging the exclusion would use reasoning more focused on moral rather than group or personal concerns. It was an open question as to whether reasoning would differ based on the group context.

Hypothesis 4 Participants would individually evaluate a bystander who challenges exclusion more positively compared to how they think the group would evaluate them, especially in intergroup contexts compared to intragroup contexts.

## METHOD

### Design and participants

The study adopted a 2 (Age Group) × 4 (Group Context) between‐participants experimental design. Participants were randomly assigned to a Group context, either intergroup or intragroup (see Table 1).

**TABLE 1:** Group membership of the excluders and victim in each group context and the number of participants randomly assigned to each context.

Group context	Excluders	Victim	No. of participants
Intergroup	British	Immigrant	80 (23.4%)
	Immigrant	British	83 (24.4%)
Intragroup	British	British	89 (26.2%)
	Immigrant	Immigrant	88 (25.9%)

*Note*: The excluders and the challenger belong to the same group within each context.

Participants were 424 British children (*N* = 205, 48.3%, range = 8–10 years, *M*_age_ = 8.97, *SD* = 0.89) and adolescents (*N* = 219, 51.7%, range = 13–15 years, *M*_age_ = 13.17, *SD* = 0.91), evenly distributed across gender (Female *N* = 209, 49.3%). The study was conducted in diverse areas of a large city in south‐eastern England with lower‐middle class participants. The final sample was comprised of 24.7% South Asian British, 17.6% White British, 17.1% Black British, 12.1% Dual‐Heritage, 9.7% European British and 6.5% other (including Arab and Japanese British), with 12.4% of the British sample withholding ethnicity information.

Participants were asked if they were British or an immigrant. Participants who self‐categorized as immigrants (*N* = 84) and were excluded from the final analyses were split across age (children, *N* = 50; adolescents, *N* = 34) and experimental condition (intergroup, *N* = 44; intragroup, *N* = 40). A non‐significant chi‐square test showed that these exclusions were random and not as a function of age or condition, *χ* ^2^(3) = 1.798, *p* = .61. A final sample of 340 participants (children, *N* = 155, *M*_age_ = 8.97, *SD* = 0.93; adolescents, *N* = 185, *M*_age_ = 13.24, *SD* = 0.92) was analysed.

Power analysis for an analysis of variance with three factors and eight groups was conducted in G*Power to determine a sufficient sample size using an Alpha level of .05, a power of 0.95 and a small to medium effect size of 0.25 (Faul et al., 2007). The required sample size was 279.

### Procedure and measures

This study was approved by the Ethics Committee of Goldsmiths, University of London. All participants received parental consent, gave assent and completed the Qualtrics survey in their school under researcher guidance before debriefing.

Participants were first asked to imagine that they were part of a group, the “British group of friends” (Killen, Rutland, et al., 2013; Mulvey & Killen, 2016), represented by age‐matched illustrations. To enhance identification with the group, participants selected a name and a symbol (a star or a lightning image) for their groups (Nesdale, 2008). Next, participants were asked to imagine another group: the “Immigrant group of friends.” Participants were presented with the following definition of immigrants: “immigrants are individuals who live in Britain but are not British since they were born in and came from other countries” (Abbott & Cameron, 2014; Cameron et al., 2006).

#### Social exclusion scenario

Next, participants read a hypothetical scenario in which either an immigrant or a British peer was excluded from a cooking club by either an immigrant or a British peer. The reason for the exclusion was ambiguous. An example scenario is (intergroup context condition): “Imagine that your group, the British group of friends, decide to form a cooking club for students who like cooking British food in your school. [Victim] from the immigrant group of friends likes cooking British food and wants to join the cooking club. [Excluder], from your group, doesn't want him/her to join the cooking club. [Excluder] shares his/her opinion with the others in the club and they agree to leave [victim] out.”

Participants then read about a peer from the excluders' group who thought that the group should have included the victim. Participants read the following: “However, [challenger], thinks that [victim] should be included in the cooking club.”

#### Expectations of peer bystander challenging

Next, we measured participants' expectations for how likely it is that the [challenger] peer will challenge the exclusion as a bystander. Participants were asked: “[Challenger] thinks that [victim] should be included in the cooking club. How likely or not likely is it that [challenger] will challenge the group?” on a 1 (really not likely) to 6 (really likely) scale (Mulvey et al., 2016). Higher numbers show higher expectations for the peer‐challenging exclusion.

#### Reasoning justifications

Participants answered an open‐ended “why?” question to justify their responses to the expectation of peer‐challenging questions. Reasoning justifications were analysed using a coding system aligned with previous SRD research (Gönültaş et al., 2022; Killen, Mulvey, & Hitti, 2013). They were coded into 4 categories under three domains: Moral (1: moral), Group (2: group dynamics, 3: group repercussions) and Personal (4: personal) (see Table 2).

**TABLE 2:** Coding, categories, themes and example items.

Coding	Categories	Themes	Example items
Moral		Fairness	“Because it is unfair”
		Equality and diversity	“Everyone should be included”
		Others' feelings, social and psychological needs	“Because it is kind” “He will be sad”
Group‐based	Group dynamics	Group Norms Group Functioning Understanding of group processes	“Because they think she'll mess up everything” “Vast majority of the group will still disagree, one person cannot change the whole group's ideas”
	Group repercussions	Social consequences Cost of Challenging	“Because if he speaks against them, he might be picked on or kicked out of the group” “Because she will be excluded”
Personal		Personal Preferences, Characteristics and Personal Opinion	“Because she wants to get involved”
Undifferentiated			“I don't really know”

Moral concerns include references to fairness, equality and, others' feelings; group concerns include references to group dynamics, and repercussions. Personal concerns include references to personal characteristics and preferences. Personal reasoning was used less than 10% for the expectations question (7.4%). They were removed from the analyses along with the statements that did not fit into the conceptual categories (9.1%). Interrater reliability was conducted on 25% of the responses by two coders. One coder was blind to the hypotheses. The analyses of agreement showed strong inter‐rater reliability (Cohen's kappa = .98).

#### Individual and perceived group evaluations of peer bystander challenging

Then participants read that the [challenger] peer does challenge the exclusion as a bystander. They were told, “[Challenger] tells the group that s/he thinks that they should include [victim] in the cooking club.” Participants were asked: (*individual evaluation*) “How okay or not okay do you think [challenger peer] was?”; (*perceived group evaluation*) “How okay or not okay does the group think [challenger peer] was?” on a 1 (really not okay) to 6 (really okay) scale (Mulvey & Killen, 2016). Higher numbers show more positive evaluations of peer challenger.

#### Intergroup contact

An adapted version of the intergroup contact measure developed by Crystal et al. (2008) was used to measure the level of intergroup contact with immigrants. The scale contained six items, (e.g., “At school, how many friends do you have who are immigrants?”) Responses range from 1 (‘none’) to 4 (‘most’), *α* = .84.

### Plan for analyses

Expectations and evaluations data were analysed using a univariate and a repeated measures ANCOVA controlling for intergroup contact. Follow‐up tests were performed using the Bonferroni correction to control for Type I errors. To test our hypotheses, an orthogonal contrast was conducted to create a Group Context dummy variable. This was done by coding the two intergroup contexts (+1) and the two intragroup contexts (−1) (see Table 1). Initially, we ran the analyses with gender as a factor and did not find any differences involving gender, so it was dropped from further analyses.

Reasoning responses were analysed using multinomial logistic regression models (see McGuire et al., 2017). The effects of Age group (children, adolescents), Group context (intergroup, intragroup context) and Expectations of peer bystander challenging (above 3.5, below 3.5) were modelled across each reasoning category. When the proceeding main effects were qualified by interaction terms and small cell sizes were observed, we conducted Fisher's exact test and follow‐up *z* tests with Bonferroni correction with multiple comparisons to investigate interactions (means are proportional percentages of reasoning).

## RESULTS

### Expectations of peer bystander challenging

We hypothesized that compared to children, adolescents would be less likely to expect a bystander peer to challenge social exclusion, especially in intergroup contexts compared to intragroup contexts. To test for H1, we conducted a 2 (Age group: children and adolescents) × 2 (Group context: intergroup and intragroup) univariate ANCOVA controlling for intergroup contact. There was a significant univariate main effect of Age group, *F*(1, 288) = 46.29, *p* < .001, $\eta_{p}^{2}$ = .138. Adolescents were less likely to expect the bystander peer to challenge the exclusion (*M* = 3.28, *SD* = 1.49), compared to children (*M* = 4.51, *SD* = 1.74), showing partial support for H1. However, the interaction effect between Age group and Group context was not statistically significant (*p* > .05). However, it is worth noting that the *p*‐value for the interaction term approached significance, *F*(1, 288) = 3.45, *p* = .064, $\eta_{p}^{2}$ = .012, suggesting a potential trend in the expected direction.

### Social and moral reasoning about bystander expectations

**FIGURE 1:**
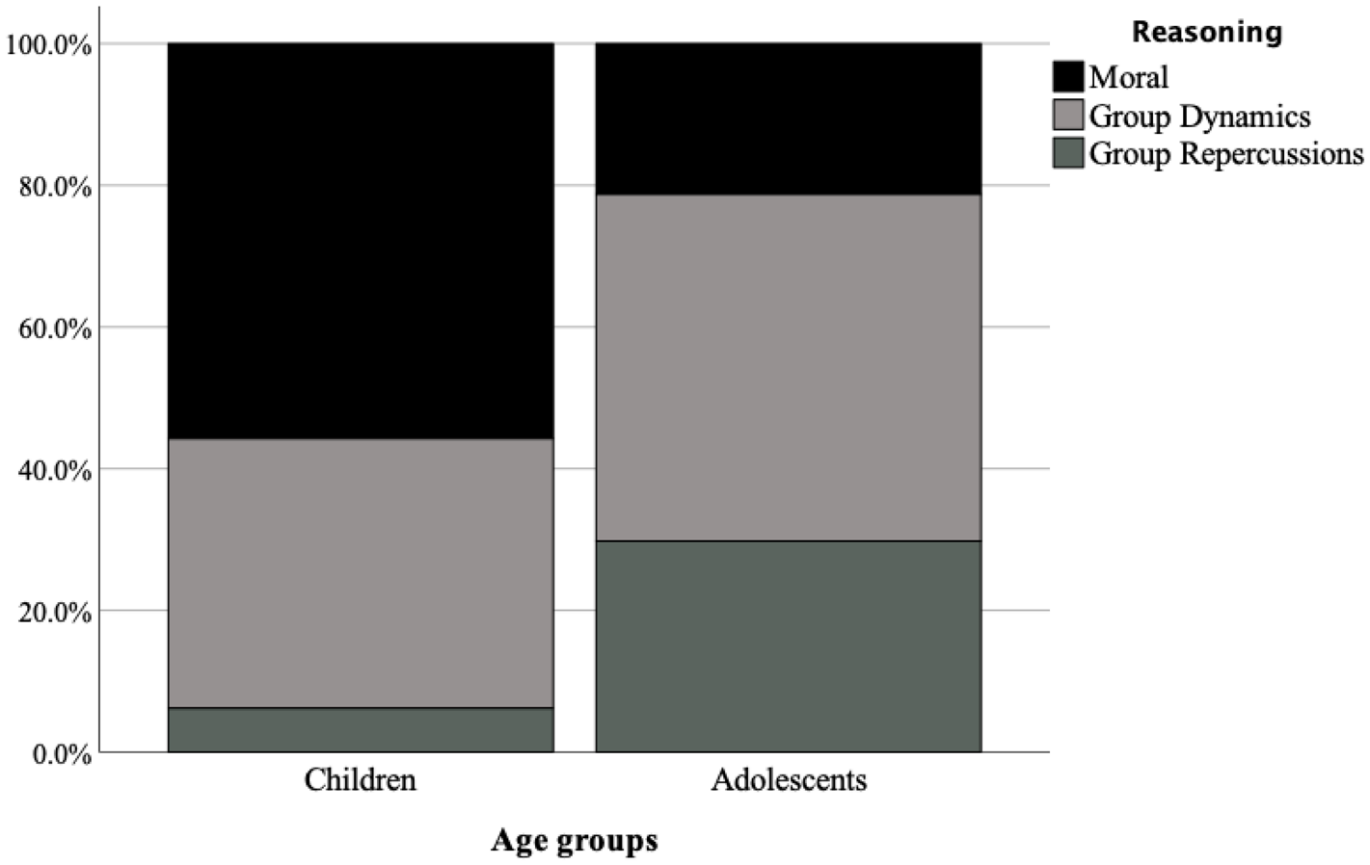
Proportions of participants' reasoning of expectations of peer bystander challenging as a function of age group.

We hypothesized that compared to children, adolescents would be more likely to refer to group and personal concerns than moral concerns when reasoning about their expectations of bystander challenging. The addition of predictors (Age Group, Group Context and Expectation) to the model led to a significant improvement in the model fit compared to the null model, (LR) *χ* ^2^(6, *N* = 253) = 104.45, Nagelkerke *R* ^2^ = .386, *p* < .001. A main effect of Age group on reasoning was found, *χ* ^2^(2, *N* = 253) = 16.33, *p* < .001. In line with H2, compared to children, adolescents were more likely to justify their expectations of a peer bystander challenging with reference to group dynamics, *β* = −0.753, *χ* ^2^(1) = 4.79, *p* = .03, Exp(*B*) = 0.471, 95% CI [0.24, 0.92] and group repercussions, *β* = −1.922, *χ* ^2^(1) = 14.26, *p* < .001, Exp(*B*) = 0.146, 95% CI [0.05, 0.39] than moral reasons (see Figure 1). There was no significant main effect of group context on reasoning, *χ* ^2^(2, *N* = 253) = 3.27, *p* = .195.

We also hypothesized that participants who report a higher expectation of the bystander challenging the exclusion would use reasoning more focused on moral rather than group or personal concerns. In support of H3, there was also a main effect of Expectation on reasoning, *χ* ^2^(2, *N* = 253) = 56.71, *p* < .001. Participants who reported low compared to high expectations of peer bystander challenging were more likely to justify this with reference to group dynamics, *β* = 2.343, *χ* ^2^(1) = 36.59, *p* < .001, Exp(*B*) = 10.412, 95% CI [4.87, 22.24] and group repercussions, *β* = 2.850, *χ* ^2^(1) = 32.766, *p* < .001, Exp(*B*) = 17.28, 95% CI [6.51, 45.85] than moral reasons.

**FIGURE 2:**
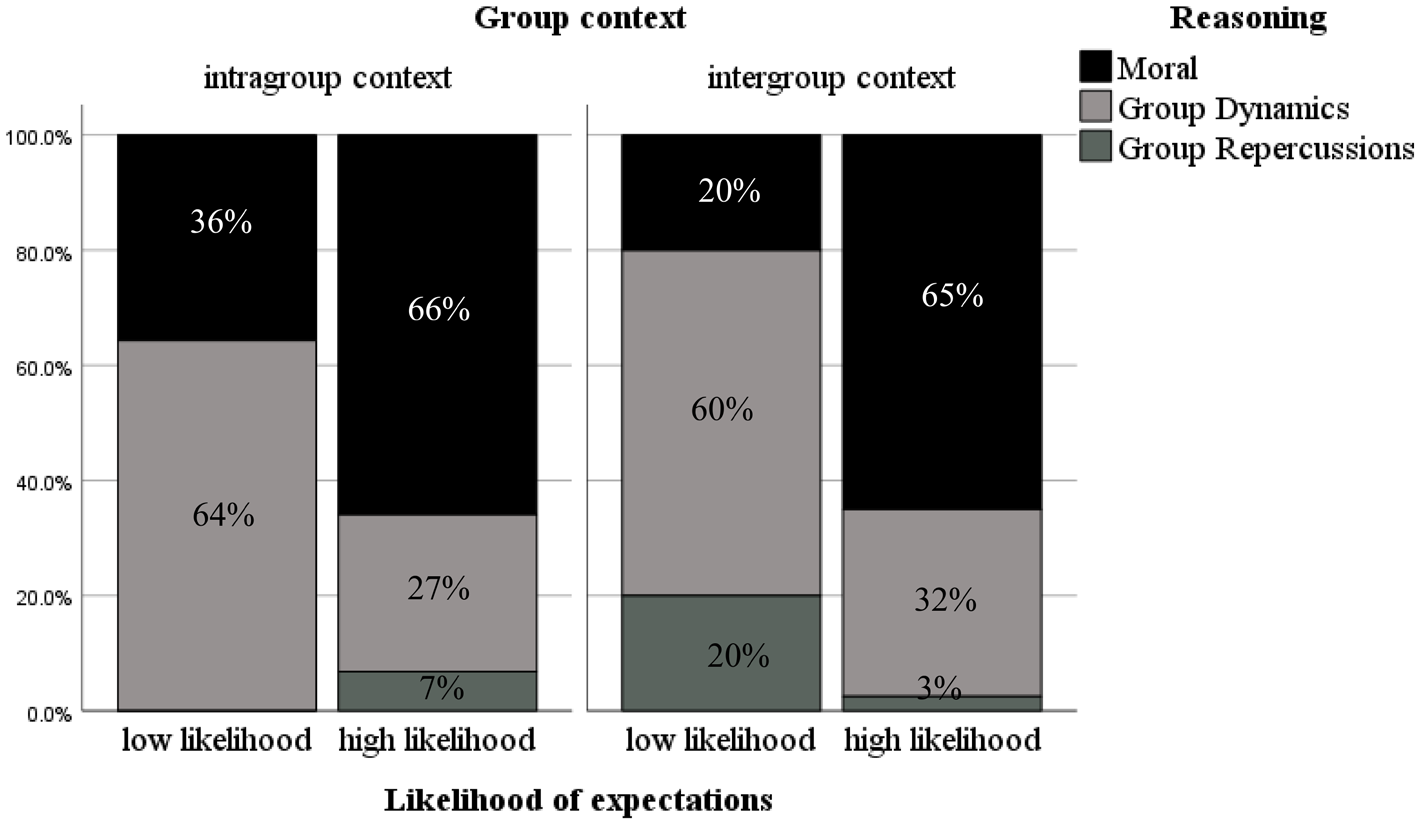
Proportions of children's reasoning of expectations of peer bystander challenging as a function of Group Context, and Expectation.

**FIGURE 3:**
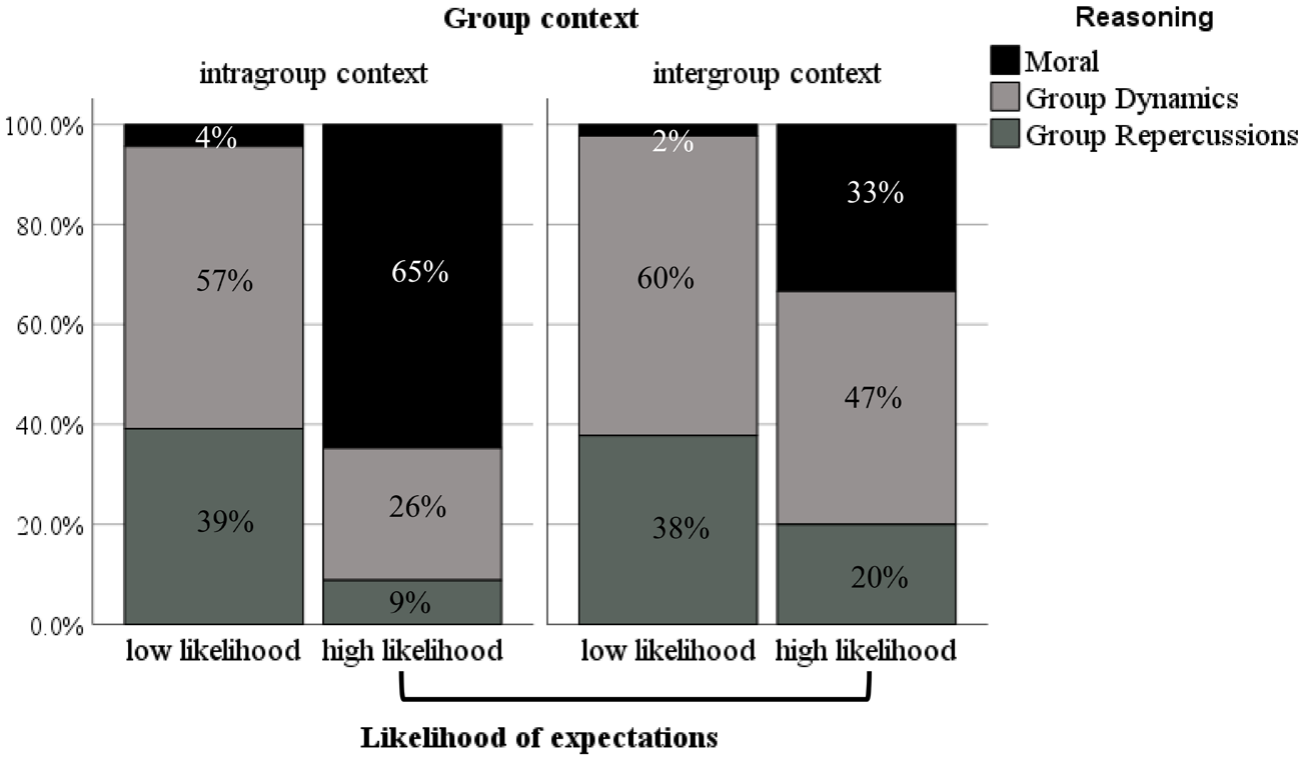
Proportions of adolescents' reasoning of expectations of peer bystander challenging as a function of Group Context and Expectation.

These effects were superseded by a significant interaction between Age group, Group Context and Expectation, which improved the fit of the model (LR) *χ* ^2^(14, *N* = 253) = 117.778, Nagelkerke *R* ^2^ = .425, *p* < .001. The reasoning differed significantly as a function of Expectation in intergroup contexts among children (Fisher's exact = 10.52, *p* = .004) and adolescents (Fisher's exact = 10.00, *p* = .005) but in different ways. First, children who reported a low expectation of peer bystander challenging in intergroup contexts made significantly greater reference to group dynamics (0.60) than moral reasons (0.20) and group repercussions (0.20) (see Figure 2). In contrast, adolescents in the intergroup contexts with low expectations were more likely to refer to group dynamics (0.60) and group repercussions (0.38) compared to moral reasons (0.2) (see Figure 3). For example, an adolescent participant evaluated their low expectations of a bystander challenging by stating, “because more people think that she shouldn't be in [the group], its 4 against 1” or, “because they might threaten her by saying they might kick her out the group.” Moreover, children who reported high expectations in intergroup contexts made significantly greater reference to moral reasons (0.65) than group dynamics (0.32) and group repercussions (0.3) (see Figure 2). For example, one child justified their high expectations for challenging by saying “because he [the excluded peer] is feeling sad.” Whereas adolescents with high expectations in intergroup contexts were more likely to justify this with reference to group dynamics (0.60) than group repercussions (0.20) and moral reasons (0.33, see Figure 3).

The reasoning also differed significantly as a function of expectation within the intragroup contexts among children (Fisher's exact = 5.75, *p* = .037) and adolescents (Fisher's exact = 36.25, *p* < .001). Children with low expectations in intragroup contexts made significantly greater reference to group dynamics (0.64) than moral reasons (0.36). There were no references to group repercussions among children (see Figure 2). Adolescents with low expectations were more likely to justify this with reference to group dynamics (0.56) and group repercussions (0.40) compared to moral reasons (0.4, see Figure 3). In contrast, when participants reported high expectations of challenging in intragroup contexts, children made significantly more reference to moral reasons (0.66) than group dynamics (0.27) and group repercussions (0.7, see Figure 2), and adolescents were also more likely to refer to moral reasons (0.65) than group dynamics (0.26) and group repercussions (0.9, see Figure 3).

Overall, the results show that children and adolescents with low expectations of challenging, especially in the intergroup contexts, used different reasoning to justify their expectation judgements. Adolescents favoured group dynamics and group repercussions reasoning rather than moral reasoning, while children did not use group repercussions reasoning instead favouring group dynamics and, to a lesser degree, moral reasoning. In contrast, both children and adolescents with high expectations of challenging, especially in intragroup contexts, prioritized moral compared to group dynamics or group repercussions reasoning.

### Individual and perceived group evaluations of bystander challenging by peer group member

**FIGURE 4:**
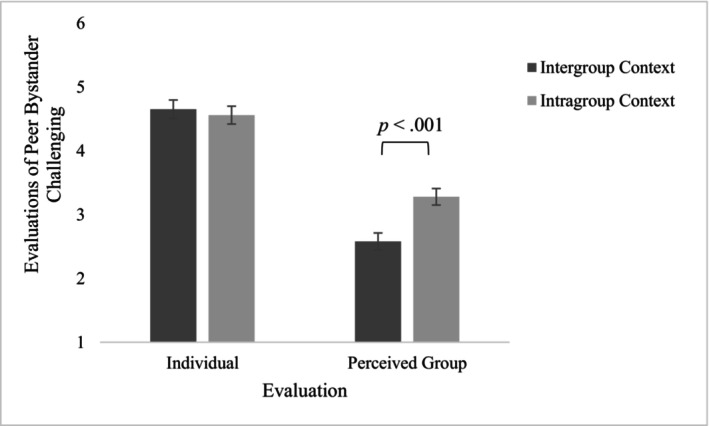
Participants' individual and perceived group evaluation of peer bystander challenging as a function of Group Context.

We hypothesized that participants would individually evaluate a bystander who challenges exclusion more positively compared to how they think the group would evaluate them, especially in intergroup contexts compared to intragroup contexts. To test for H4, A 2 (Age Group: Children and Adolescents) × 2 (Group context: Intergroup and Intragroup) × 2 (Evaluation: Individual Evaluation and Perceived Group Evaluation) ANCOVA with repeated measures on the last factor was performed, controlling for intergroup contact. In line with H4, there was a main effect of Evaluation, *F*(1, 283) = 23.64, *p* < .001, $\eta_{p}^{2}$ = .077. Participants individually evaluated the bystander challenging the social exclusion as more acceptable (*M* = 4.60, *SD* = 1.70) compared to how they perceived the peer group would evaluate them (*M* = 2.94, *SD* = 1.67). In support of H4, there was also a significant interaction between Evaluation and Group Context, *F*(1, 283) = 11.77, *p* < .001, $\eta_{p}^{2}$ = .040. Pairwise comparisons showed that participants thought the peer group would evaluate the bystander challenging exclusion as more acceptable within intragroup contexts (*M* = 3.28, *SD* = 1.72) compared to intergroup contexts (*M* = 2.58, *SD* = 1.53, *p* < .001, $\eta_{p}^{2}$ = .055). There was no difference in their individual evaluations across the two contexts (*M*_intragroup_ = 4.56, *SD* = 1.73; *M*_intergroup_ = 4.65, *SD* = 1.68, *p* = .760, see Figure 4).

## DISCUSSION

This study explored children's and adolescents' expectations, evaluations and reasoning about a bystander who challenges the group‐based social exclusion of immigrant or non‐immigrant peers in different group contexts. Our results provide novel insight on developmental differences in how children and adolescents think and reason about bystander peers who challenge the social exclusion of immigrant peers. Compared to children, adolescents were less likely to expect a peer bystander who wanted to challenge social exclusion to do so.

This finding is in line with the SRD approach, which would expect that adolescents to consider group‐related concerns (e.g., group dynamics and the consequences of challenging group norms, namely group repercussions) more than children when evaluating peer group social exclusion (Mulvey et al., 2016; Mulvey & Killen, 2016; Palmer & Abbott, 2018). This is because adolescents compared to children are known to have a better understanding of group dynamics and intergroup processes alongside greater social perspective taking (Im‐Bolter et al., 2016; Killen & Rutland, 2011; Vetter et al., 2013). Indeed, in the present study we found that adolescents compared to children justified their low expectations of peer bystander challenging exclusion with reference to group‐based reasoning, especially group repercussions. However, our prediction for an interaction between age group and group context on expectations of challenging was not supported, although there was a trend in the expected direction. This might be because both intergroup and intragroup contexts in this study were peer group contexts in which a peer was excluded by other peers. In both contexts, maybe the presence of peers within an group was enough to prompt consideration of peer group dynamics (i.e., will the bystander peer be perceived as disloyal within the peer group?) when forming expectations about whether the bystander will challenge.

The novel reasoning findings also revealed that in intergroup contexts, adolescents with low expectations of challenging favoured group dynamics and group repercussions reasoning rather than moral reasoning, while children did not use group repercussions reasoning. This aligns with previous research by Mulvey et al. (2016), which suggests that awareness of intergroup processes and group repercussions increases with age (Mulvey et al., 2016). Even adolescents who had high expectations of challenging in intergroup contexts significantly used group dynamics and group repercussion justifications while they referred more to moral reasons in intragroup contexts.

This study also explored participants' individual and perceived group evaluations of peer bystander challenging. As expected, participants individually supported challenging the exclusion more than they thought the group would. Importantly, contextual differences were found in evaluations of peer challenging across intergroup and intragroup contexts. Youth individually supported peer‐challenging exclusion regardless of group context. This is in line with research showing that youth are supportive of peers who challenge unfair resource allocation (Killen, Rutland, et al., 2013). Participants, however, were less likely to think that the peer group would be supportive of peer challenging in intergroup contexts compared to intragroup contexts. This finding supports one of the main tenets of the SRD approach: that the importance of group‐related factors (e.g., group identity, group loyalty and group norms) becomes more salient in intergroup contexts such that, when evaluating bystander challenging, youth pay more attention to group compared to moral concerns (Rutland et al., 2010). This finding also shows that youth can and do differentiate intergroup exclusion from intragroup exclusion. This is in line with the SRD approach, which distinguishes intergroup exclusion, which stems from discrimination and prejudice, from other forms of exclusion, that is, intragroup and interpersonal exclusion (Killen, Mulvey, & Hitti, 2013; Killen & Rutland, 2011). This highlights the fact that reducing intergroup exclusion requires a different approach (Killen, Mulvey, & Hitti, 2013). A greater focus on group dynamics and intergroup processes (i.e., group norms and intergroup relations) is therefore required when developing interventions to support inclusive school environments, including anti‐bullying interventions that might focus on enhancing peer‐based social inclusion and reducing social exclusion as a specific form of anti‐bullying.

While the current study provides novel findings, future research should also investigate participants' bystander challenging behaviour to explore how they would react in real life rather than in hypothetical situations (see Yüksel et al., 2021). Second, the current study is cross‐sectional in nature and cannot tell us about the developmental changes over time, only developmental differences. Future longitudinal studies would help to capture a complete developmental picture, highlighting developing understanding of bystander challenging across different contexts. Third, the present study only examined British majority‐status youths' evaluations and lacked a minority‐status immigrant sample. Minority‐status participants' perspectives remain understudied but are needed to better understand how to make diverse school environments more inclusive. Recent findings suggest that, compared to majority‐status national youth, minority‐status immigrant youth respond differently to intergroup social exclusion in adolescence (Palmer et al., 2022). A better understanding of how we can increase prosocial bystander responses among majority‐status youth and improve support for minority‐status youth is needed to improve school‐based programmes, which are found to be less effective in diverse settings (Evans et al., 2014).

The findings have practical implications for school interventions aimed at reducing exclusion by promoting bystander challenging. They indicate interventions should adopt different approaches for different age groups, and with a focus on different factors of influence. While a morally focused intervention model might work for children, interventions for adolescents might require a greater focus on group‐related factors. For example, this could include encouraging adolescents to think critically about exclusive group norms (e.g., expectations for groups not to include others), which often discourage bystander challenging (Palmer et al., 2015), while also making adolescents more focused on the psychological and social consequences of standing up to peers who exclude immigrants.

This study shows how group context matters for youth's expectations, evaluations of and reasoning about peer bystander challenging towards immigrant social exclusion. It showed for the first time how children and adolescents differ in their expectations, evaluates and reasoning about peer bystander challenging in intergroup compared to intragroup contexts. Additionally, the research underscores the importance of considering the broader implications of immigrant‐based social exclusion and the role of bystanders in childhood and adolescence. Given the current context of the United Kingdom and many other countries facing similar issues, this study highlights the need for targeted interventions that address these social dynamics. By focusing on immigrant contexts, we emphasize the critical need to foster inclusive attitudes and behaviours among youth to combat social exclusion and its detrimental effects on marginalized groups.

## AUTHOR CONTRIBUTIONS

**Ayşe Şule Yüksel:** Conceptualization; investigation; writing – original draft; methodology; validation; visualization; writing – review and editing; software; formal analysis; project administration; data curation; resources. **Sally B. Palmer:** Conceptualization; supervision; writing – review and editing. **Eirini K. Argyri:** Data curation; writing – review and editing. **Adam Rutland:** Conceptualization; investigation; methodology; supervision; writing – review and editing.

## CONFLICT OF INTEREST STATEMENT

The authors declare that they have no conflict of interest.

## Data Availability

The data that support the findings of this study are available from the corresponding author upon reasonable request.
